# Subclinical inflammation associated with monosodium urate crystal deposition: a cellular and proteomic study in synovial fluid

**DOI:** 10.3389/fimmu.2026.1888214

**Published:** 2026-08-06

**Authors:** Mariano Andrés, María-Luisa Peral-Garrido, Samanta Ortuño-Miquel, Rocío Caño, Silvia Gómez-Sabater, Alejandra Bermúdez-García, Teresa Lozano, Miguel Perdiguero, Elena Caro-Martínez, Ruth Sánchez-Ortiga, Carolina Ruiz-García, Rubén Francés

**Affiliations:** 1 Dr. Balmis General University Hospital, Alicante Institute for Health and Biomedical Research (ISABIAL), Alicante, Spain; 2 Miguel Hernandez University of Elche, San Juan de Alicante, Spain; 3 Vinalopó University Hospital, Elche, Spain; 4 HACLE San Vicente Hospital, San Vicente del Raspeig, Spain; 5 Campoamor Health Center, Alicante, Spain; 6 CIBERehd, Carlos III Health Institute, Alicante, Spain

**Keywords:** asymptomatic hyperuricemia, chemokines, crystals, gout, inflammatory proteomics, leukocytes

## Abstract

**Background/purpose:**

Subclinical inflammation linked to monosodium urate (MSU) crystals in gout and asymptomatic hyperuricemia (AH) remains poorly understood. We aimed to assess the cellular and inflammatory proteomic profiles in synovial fluid (SF) associated with MSU crystals, as observed by ultrasound or microscopy.

**Methods:**

100 participants with AH or intercritical gout underwent clinical assessment, blood testing, musculoskeletal ultrasound, and SF aspiration. 81 samples were available for assessment. SF leukocyte counts and the inflammatory proteome from cell lysates were studied. Samples were compared based on ultrasound deposition (double-contour sign, tophi, or aggregates) and microcrystal deposition (MSU, CPP, no crystals). The analyses included protein expression comparisons, paired SF-serum correlations, and machine-learning predictive models development.

**Results:**

Leukocyte counts were higher in samples with MSU crystals [n=5] than in samples with CPP crystals [n=11] or no crystals [n=65]. A third of the MSU crystals were found intracellularly. MSU-containing samples showed several inflammatory proteins upregulated, including OSM, ADA, MCP3, CXCL-1, CXCL-6, and TNFSF14. CXCL-1 was preponderant in the predictive models. Ultrasound deposits were not associated with differences in leukocytes or proteome expression. Pro-inflammatory chemokines showed direct relationships at paired SF-sera correlations.

**Conclusion:**

Despite its low prevalence in our sample, MSU crystal deposition was associated with subclinical inflammation characterized by elevated leukocyte counts and a distinctive inflammatory proteome in SF cells. Conversely, ultrasound deposits were not predictive of differences in SF inflammatory features. Most subclinical inflammation appears localized to the joint, but the parallel expression in the chemokine system requires further investigation to determine its relevance.

## Highlights

- Subclinical inflammation occurs in asymptomatic hyperuricemia and gout between clinical flares.
- MSU crystals are associated with higher leukocyte counts and an inflammatory synovial proteome.
- Ultrasound deposits may not reflect synovial inflammatory activity.

## 1 Background

Gout is the most prevalent inflammatory arthritis worldwide, affecting up to 5% of adults (1). Caused by the pathological formation of monosodium urate (MSU) crystals in joints and soft tissues after a prolonged rise in urate levels, its hallmark clinical presentation includes painful, recurrent flares, which sometimes resolve spontaneously within a few weeks. In the early stages, months or even years may pass without an episode, but if hyperuricemia remains unmanaged, the burden of MSU crystals and the frequency of flares increase, potentially resulting in persistent arthritis. Up to 40% of patients fail to meet urate targets in specialized settings (2, 3), with worse figures in general care (4, 5). Insufficient or absent management of gout leads to tophi formation, joint damage, impaired quality of life, high risk of cardiovascular and renal comorbidities, and increased chances of mortality (6). In light of the rising global prevalence of gout (7), gout is emerging as a major public health concern.

There have been substantial insights into the mechanism behind the acute inflammatory response to MSU crystals, which clinically manifests as a gout flare. Innate immune cells recognize MSU crystals as a danger-associated molecular pattern (8). The crystal promotes the activation of the NACHT, LRR, and PYD domains-containing protein 3 (NLRP3) inflammasome, which in turn cleaves pro-caspase-1 to caspase-1, leading to the activation of interleukin (IL)-1β (IL1B) and IL18. In parallel, caspase-1 processes gasdermin-D, creating membrane pores that facilitate the secretion of cytokines and nuclease enzymes. DNA fragmentation, ion fluxes, and fluid imbalance ultimately lead to cell death (9). Neutrophils predominate in synovial fluid (SF) during gout flares. Reaching the synovial cavity, they secrete proteases that cleave IL-1α and IL1B precursors released from local dying cells, thereby extending the inflammatory process (10). However, gout flares are self-limited, probably due to the entrapment of crystals and inflammatory mediators by chromatin-based neutrophil extracellular traps (or NETs), a shift toward anti-inflammatory cytokines such as transforming growth factor-β (TGFB) or IL10, or the lack of a subsequent adaptive immune response (11, 12). However, inflammation is not completely resolved; a subclinical inflammation related to MSU crystals persists.

Between gout flares, untreated gout is associated with persistent systemic inflammation (13, 14). However, it is difficult to differentiate the effect of crystal deposits from the intrinsic inflammatory potential of hyperuricemia (15). In joints and soft tissues, cumulative data indicate persistent inflammation during intercritical gout. Pascual and colleagues reported elevated leukocyte counts in SF containing MSU crystals (16), lowered by administering colchicine (17), with 22.6% of cells showing intracellular crystals, suggesting active phagocytosis (18). Leukocytes and MSU crystals are lower in patients who achieved urate <6mg/dL under treatment (19). Tophi sometimes exhibit a reddish appearance on inspection, especially when located in superficial areas. CD68^+^ cells expressing IL1B and TGFB are found surrounding crystal packages in a granuloma-like appearance (20). Ultrasound shows a positive power-Doppler (PD) signal in crystal deposits in 67.0% of patients (grade 2–3 in 30.1%) during the intercritical stage (21). The predominant location is the metatarsophalangeal (MTP)-1 joint (22). Similar data on persistent inflammation is available for asymptomatic hyperuricemia (AH) with MSU crystal deposition. There is an active local PD signal in 22.8-36.7% of people (23, 24), elevated leukocyte counts in SF (25), and higher serum levels of IL6, IL8, miRNA-155, calprotectin, and high-sensitivity C-reactive protein (hsCRP) (26, 27).

Despite cumulative evidence on MSU-related subclinical inflammation, the underlying mechanism remains unclear. Understanding subclinical inflammation may offer insights into preventing recurrent flares and other MSU crystal-related consequences, such as disability (28), bone erosions (29), or cardiovascular disease (30). Accordingly, our study examined SF cellularity and the inflammatory proteome associated with MSU crystal deposition in hyperuricemic patients without inflammatory symptoms. We also assessed the impact of defining crystal presence using ultrasound or microscopy.

## 2 Methods

This cross-sectional study is part of a larger project that comprehensively assessed the inflammatory status across the uricemia spectrum (27, 31). The study was conducted at a Spanish tertiary academic center, after approval from the ethics committee of Alicante-General Hospital (ref. PI2018/044) and the responsible research office of Miguel Hernández University of Elche (ref. ADH.SPU.MNAC.MLPG.23). All participants received information about the study and voluntarily signed a consent form.

### 2.1 Participants and data collection

Participants at any stage of the uricemia spectrum (normouricemia, AH, intercritical gout) were recruited from Internal Medicine, Cardiology, Nephrology, Endocrinology, Rheumatology, and Primary Care. Here, we focused only on individuals with hyperuricemia (defined as a serum urate (SU) level of ≥7mg/dL at the last test) and available SF analysis. Per protocol, no SF analysis was performed in those with normouricemia. Hyperuricemic participants could have AH (reporting no background indicating flares) or intercritical gout (according to the ACR-EULAR 2015 classification criteria (32)). Current use of ULT, colchicine, or immunosuppressants was a reason for exclusion, as was having another concurrent inflammatory rheumatic disease.

Participants’ medical records were reviewed, and they completed clinical questionnaires, physical examination, and blood testing. Biomarkers included urate (in mg/dL, by uricase), hsCRP (in mg/dl, by immunoturbidimetry), serum amyloid A (SAA, in mg/L, by nephelometry), erythrocyte sedimentation rate (ESR, in mm/h), total leukocyte count (per mm3), and neutrophil to lymphocyte ratio (NLR). Part of the participants’ sera was used to measure inflammatory cytokines and proteins associated with pyroptosis and neutrophil activity (27). Serum remnants were stored at -80°C.

Next, an expert sonographer (MA), blinded to clinical and laboratory features, scanned knees, ankles, MTP-1, and MTP-2 joints. Patients lie supine, extending their knees for scans of the articular recesses, flexing their knees to 90 degrees for tibiotalar and MTP scans, and achieving maximal knee flexion to assess the condylar cartilage. All regions were examined across the width of their dorsal aspects; for MTP-1, both dorsal and medial aspects were explored. The transducer was applied both transversely and longitudinally to the regions of interest. We used VINNO E30 ultrasound equipment (Suzhou, PRC) with a multifrequency linear transducer (X6-16L model, 12–14 MHz). For cases with joint effusion, joint-guided puncture was offered. If multiple joints showed effusion, the decision on which joint to aspirate was at the sonographer’s discretion.

At the aspirated joint, the sonographer recorded the presence of gout lesions—double-contour (DC) sign, tophi, and aggregates —following the OMERACT definitions (33). At least a grade 1 (probable lesion) of any lesion was required to establish its presence.

Immediately after aspiration, two trained observers (MA and MLPG), blinded to clinical and laboratory features or ultrasound findings, respectively, simultaneously analyzed SF using a compensated polarized-light microscope with two viewing stations (Olympus BX41, Olympus, Shinjuku, Tokyo, Japan). Samples were examined for microcrystals (MSU and calcium pyrophosphate – CPP), and these were classified based on shape, birefringence, and elongation (34), and recorded as intra- or extracellular. Simultaneously, leukocytes per mm³ were counted using a Neubauer chamber (16). Remnants of SF samples were immediately resuspended in phosphate-buffered saline and centrifuged at 3500rpm for 10min; pellets were extracted and stored at -80°C. Hyaluronidase was not applied.

The SF proteome was studied using Olink Proximity Extension Assay (PEA). Due to the high viscosity and diverse volumes, protein expression was studied in cell extracts. SF-derived cells were lysed using RIPA Buffer (Millipore) following the manufacturer’s instructions. Total protein concentration was determined using the Bradford assay, and samples were normalized to 0.5µg/µL. Subsequently, 1µL of the protein lysate was used for PEA. The expression of 92 proteins (target 96 inflammation panel, full list available in the **Supplementary Material**; **Supplementary Table 1**) was studied and quantified as the normalized protein expression (NPX), as indicated by the manufacturer.

For participants whose SF samples passed quality controls and yielded proteomic results (n=60), we intended to conduct a paired serum proteome assessment using Olink PEA technology; this was possible in 28 cases. For serum samples, 1µL was directly used in the PEA assay without prior processing, using the same target 96 inflammation proteomic panel.

### 2.2 Statistics

Statistical analyses were performed using SPSS v25 (IBM, Armonk, NY), Prism v10.6.1 (GraphPad Software, Boston, MA), and R software v4.5.0 (R Core Team, https://www.R-project.org/) with the following packages: dplyr (v1.1.4) – data curation -, nortest (v1.0-4) – statistical testing -, ggplot2 (v3.5.2) – graph plotting -, limma (v3.64.4) – protein expression comparisons - and EnhancedVolcano (v1.22.0) - volcano plotting. The classification model was implemented in Python (v3.12.9), utilizing several key libraries including pandas (v2.2.3) for data manipulation, scikit-learn (v1.6.1) for machine learning utilities, XGBoost (v3.0.0), LightGBM (v4.6.0), and CatBoost (v1.2.8) for gradient boosting algorithms, as well as NumPy (v2.0.1) for numerical operations, and Matplotlib (v3.10.0) and Seaborn (v0.13.2) for data visualization.

Statistical significance was set at <0.050, and the Bonferroni correction was applied to adjust for multiple comparisons, as appropriate.

#### 2.2.1 Descriptive statistics and bivariate comparisons

Samples were classified according to ultrasound and polarized light microscopy (PLM) findings. Ultrasound results were used to characterize the aspirated joint based on the presence of deposits, understood as any grade of DC sign, tophi, or aggregates (33). PLM findings were used to classify joints as “MSU”, “CPP”, or “no crystals”. In line with our primary objective, joints with deposits and with MSU crystals were our groups of interest.

Leukocyte counts and the percentage of phagocytosis (calculated as the ratio of intracellular crystals to the total crystals in the sample) are presented as medians and interquartile range (IQR).

Data were analyzed using nonparametric statistical methods, as distribution normality was not met according to the Kolmogorov–Smirnov test. Group comparisons were performed with the Kruskal–Wallis H test, and bivariate and *post hoc* analyses were conducted using the Mann–Whitney U test with Bonferroni correction for multiple comparisons. Correlations between SF leukocytes and serum variables were evaluated using Spearman’s ρ due to the nonparametric nature of the data. Categorical variables were expressed as absolute frequencies and percentages.

Bivariate comparisons for individual protein expression were conducted within study groups using Mann-Whitney U and Kruskal-Wallis H tests, as necessary. The Bonferroni correction was applied to adjust for multiple comparisons.

#### 2.2.2 Multivariate analyses and volcano plots

The limma package in R was used to identify differentially expressed biomarkers among the study groups. Values below the detection limit were imputed as zero. For the statistical analysis, a design matrix without an intercept was constructed, allowing each category of the clinical factor to be explicitly modeled and facilitating direct comparisons through linear contrasts between groups. Linear models were fitted using the lmFit function, the defined contrasts were applied, and the eBayes function was used to apply an empirical Bayes adjustment. This stabilizes variance and improves the estimation of statistics, especially when sample sizes are small. Proteins with an adjusted *P* < 0.050 were considered significantly expressed. Comparisons were visualized using volcano plots generated with ggplot2 and EnhancedVolcano, highlighting significant proteins in red and annotating the most relevant ones for interpretation. The Bayesian adjustment in limma helps ensure the reliability of these red points, thereby reducing the likelihood of highlighting biomarkers due to chance or noise.

#### 2.2.3 Classification models

Later, we investigated whether leukocyte count and protein expression in SF could be used to construct accurate predictive models and differentiate samples within study groups. We implemented a diverse set of algorithms along with different feature selection strategies. Models’ performance was evaluated on the test set using standard classification metrics. The methodology and complete evaluation of model classification is provided in full in the **Supplementary Material** (**Annex I and Spreadsheets 1, 2**). Accordingly, we selected the following models: For *deposits*, KNN with lasso-10 (kappa 0.67, AUC 0.65, accuracy 0.88), LR with RFE-20 (kappa 0.54, AUC 0.73, accuracy 0.81), and XGBoost with lasso-20 (kappa 0.59, AUC 0.80, accuracy 0.81). For *microcrystals*, models were AdaBoost with RFE-20 (kappa 0.78, AUC 0.91, accuracy 0.94) and RF with RFE-5 (kappa 0.78, AUC 0.91, accuracy 0.88). The individual proteins contributing to the selected models are presented separately for each study group in the Results section.

#### 2.2.4 Synovial fluid-serum correlations with protein expression

The correlation between individual relative protein expression in 28 participants with paired SF and sera was calculated using Spearman’s ρ coefficient with their p-values. Results are represented using correlation heatmaps for variables with significant findings (p<0.050).

## 3 Results

**Figure 1:**
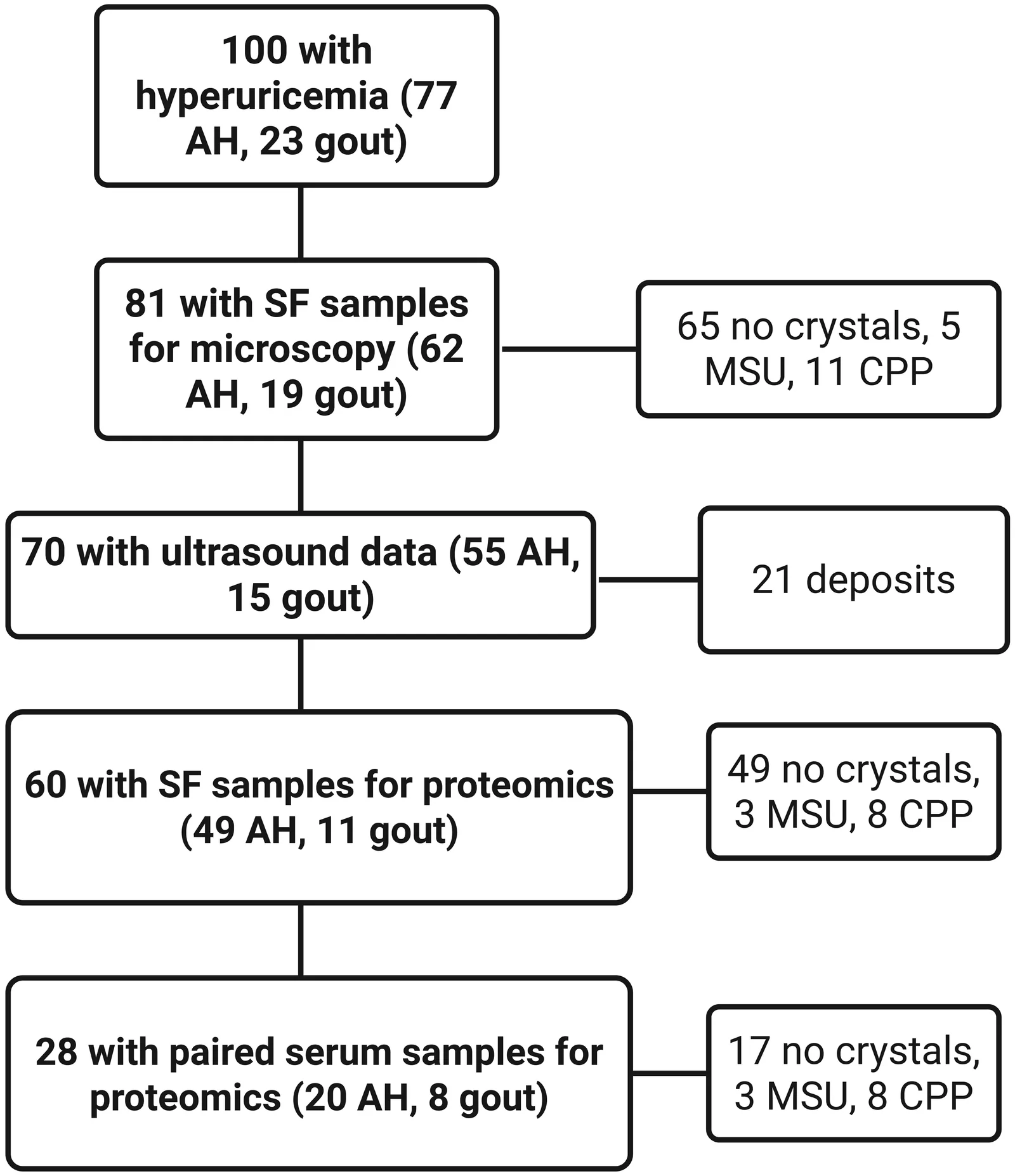
Flowchart of synovial fluid samples available for study across different stages, with their clinical, sonographic, and microscopy characteristics. AH, asymptomatic hyperuricemia; MSU, monosodium urate; CPP, calcium pyrophosphate. Created in https://BioRender.com.

Of 102 recruited participants, two were excluded due to a seronegative arthritis diagnosis and the recent initiation of febuxostat, respectively. Of the remaining 100 participants, 81 (81.0%) had SF samples available for PLM (**Figure 1**). They were primarily middle-aged men with advanced comorbidity (**Table 1**). 62 had AH, and 19 had intercritical gout. Groups were comparable with respect to clinical and laboratory characteristics (**Supplementary Table 2**). No participants were on glucocorticoids at the time of the assessment.

**Table 1:** Characteristics of participants with available synovial fluid assessment (n=81).

Variables	N (%)^a^
Demographics and clinical data	
Age, in years, median (IQR)	63.0 (49.5-76.0)
Men	59 (72.8)
White	73 (90.1)
Hyperuricemic condition	
AH	62 (76.5)
Gout	19 (23.5)
Comorbidity index, median (IQR)	4.0 (2.0-5.0)
Hypertension	57 (70.4)
Diabetes mellitus	18 (22.2)
Dyslipidemia	59 (72.8)
History of smoking	49 (60.5)
Established cardiovascular disease	26 (32.1)
Body mass index, in kg/m^2^, median (IQR)	30.6 (27.8-34.2)
Laboratory data	
Urate, in mg/dL, median (IQR)	7.5 (6.9-8.6)
eGFR, in mL/min/1.73m^2^, median (IQR)	73.4 (54.8-93.9)
hsCRP, in mg/dL, median (IQR)	0.3 (0.2-0.8)
ESR, in mm/h, median (IQR) [n=78]	21.0 (10.0-33.3)
SAA, in ng/mL, median (IQR) [n=80]	5.0 (4.0-11.0)
Total leukocytes, per mm^3^, median (IQR)	8090.0 (6600.0-9760.0)
Neutrophil:lymphocyte ratio, median (IQR)	2.4 (1.7-3.1)
Ultrasound data (n=70)	
Index joint	
Knee	59 (84.3)
MTP1	8 (11.4)
Ankle	3 (4.3)
Gout lesions	
Deposits	21 (30.0)
Double-contour sign	8 (11.4)
Tophi	7 (10.0)
Aggregates	7 (10.0)

IQR, interquartile range; AH, asymptomatic hyperuricemia; eGFR, estimated glomerular filtration rate, by CKD-EPI equation; hsCRP, high-sensitivity C-reactive protein; ESR, erythrocyte sedimentation rate; SAA, serum amyloid A; MTP, metatarsophalangeal.

a Unless stated otherwise.

Ultrasound revealed deposits in 30.0% (21/70) of aspirated joints, predominantly knees (**Table 1**), with the DC sign standing out as the most prevalent finding.

Under PLM, MSU crystals were detected in 5 samples (6.2%; 4 with gout, 1 with AH), and CPP crystals in 11 (13.6%; 9 with AH, 2 with gout). The median concentration of MSU crystals was 50/mm^3^ (IQR 0-500) and of CPP, 25/mm^3^ (IQR 0-245). The remaining 65 samples showed no crystals (80.2%; 52 with AH, 13 with gout). No cases with both crystals were detected.

### 3.1 Higher leukocytes in SF with MSU crystals

The median SF leukocyte count in the entire sample was 50/mm^3^ (IQR 20-90), but was significantly higher in samples with MSU crystals (640/mm^3^, IQR 245-875) compared to CPP (50/mm^3^, IQR 40-90) or no crystals (40/mm3, IQR 20-70) (among the three groups: p=0.018; MSU vs. no crystals: p=0.016; MSU vs. CPP: p=0.056). The median percentage of intracellular crystals was 38.5% (IQR 0-61.8%) in MSU samples and 8.3% (IQR 0-53.3%) in CPP samples (p=0.861).

**Figure 2:**
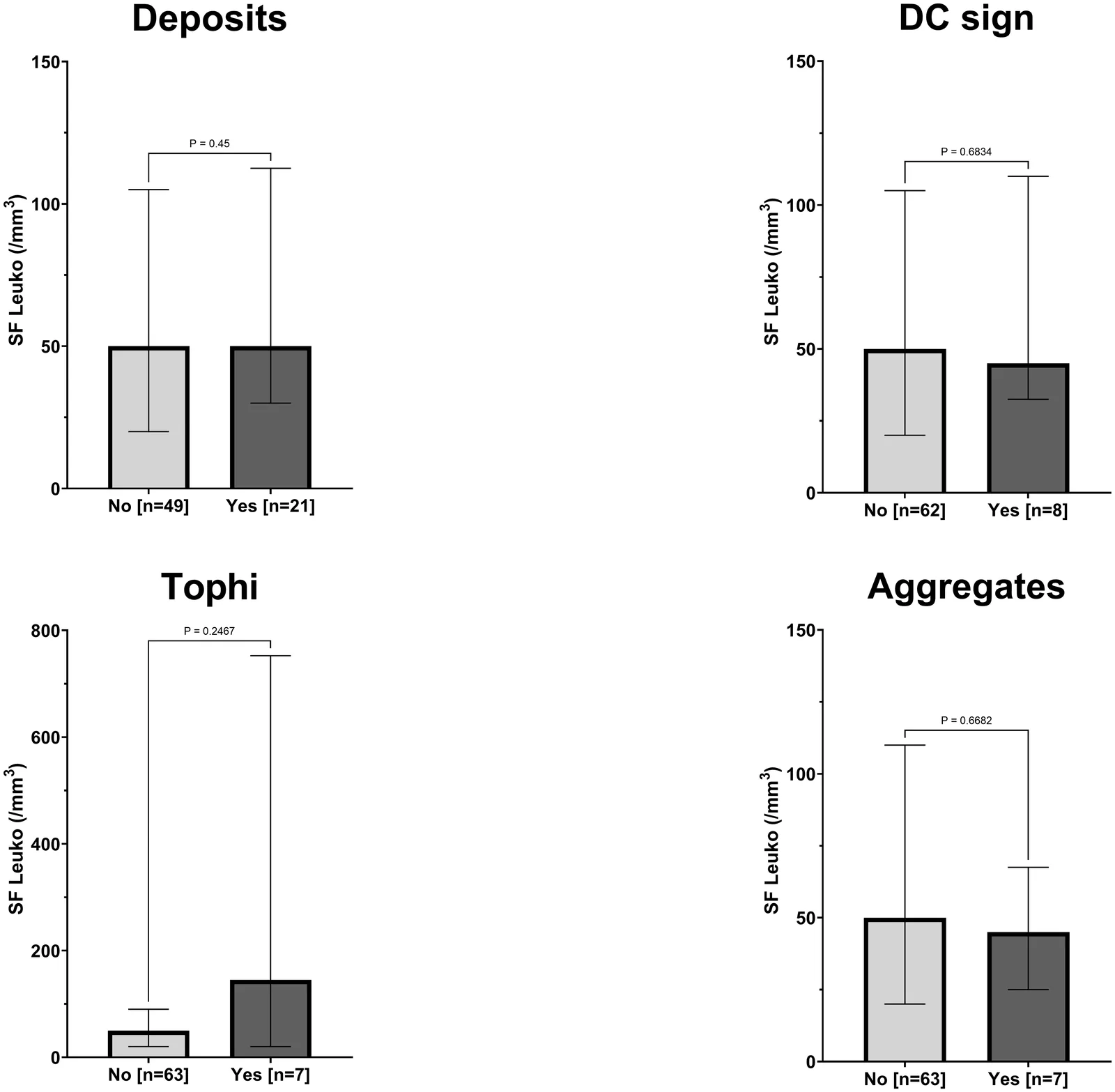
Leukocyte levels in synovial fluid samples according to ultrasound deposits and individual lesions at the aspirated joint. SF, synovial fluid; DC, double contour.

**Figure 2** illustrates the distribution of leukocytes according to different ultrasound characteristics, with no differences observed regarding deposits or their individual features.

SF leukocyte count showed no correlation with serum urate (rho=-0.05, p=0.654), hsCRP (rho=-0.11, p=0.340), ESR (rho=+0.02, p=0.875), SAA (rho=-0.01, p=0.950), total leukocytes (rho=+0.01, p=0.915), or NLR (rho=-0.04, p=0.708).

### 3.2 Distinctive inflammatory proteome with MSU crystals, but not with ultrasound deposits

Sixty samples, all passing quality assessments, were available for proteomic analysis (49 with AH, 11 with gout). Ultrasound showed crystal deposits in 18 (30.0%), while PLM showed MSU crystals in 3 (5.0%), CPP crystals in 8 (13.3%), and no crystals in 49 (81.7%).

Scant differences were noted in protein expression according to ultrasound deposits. In a bivariate comparison, MCP2 and IL4 were modestly downregulated in samples without deposits (**Supplementary Figure 1**). However, these differences were not confirmed by the volcano plot (data in **Supplementary Table 3**).

**Figure 3:**
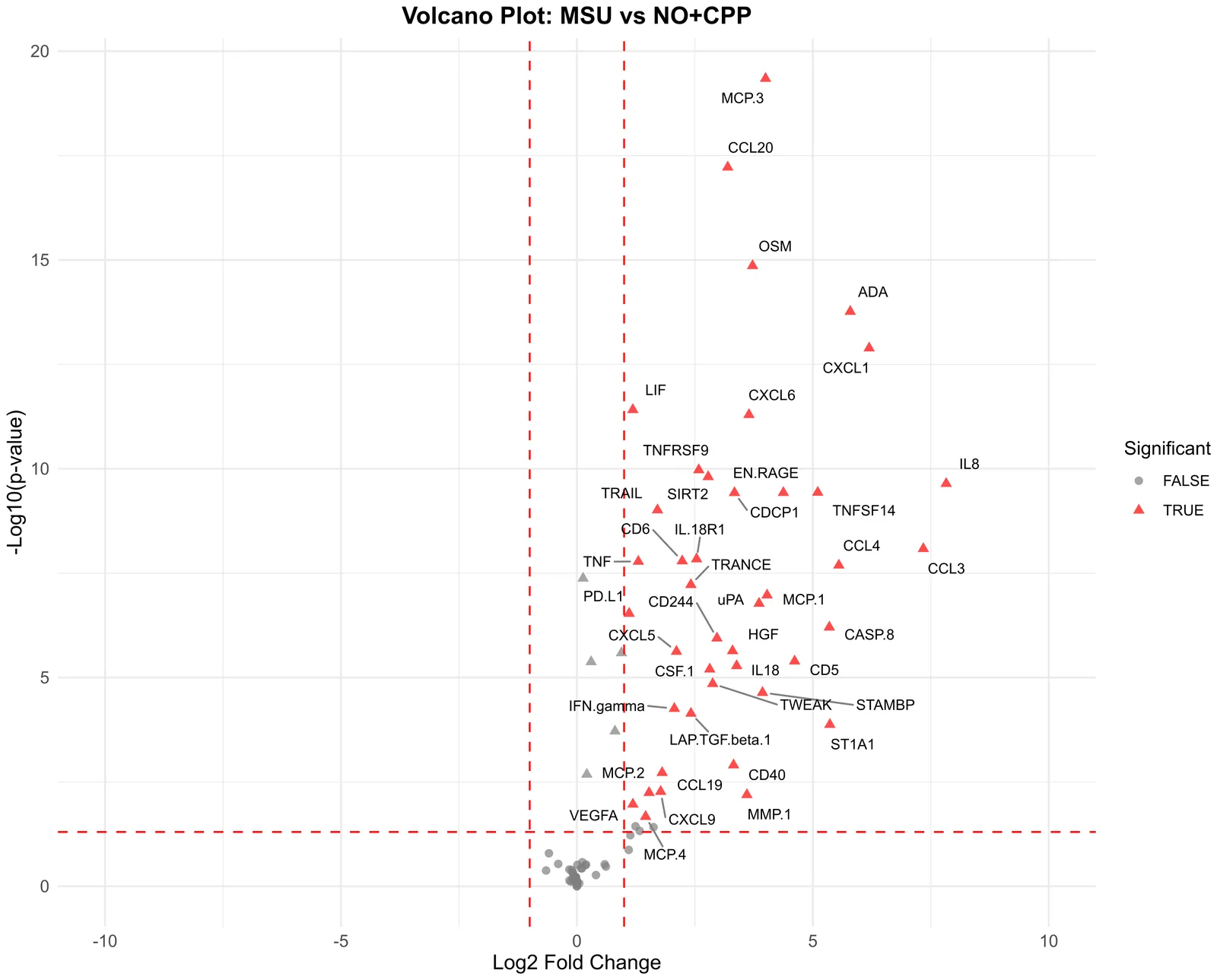
Volcano plot of differentially expressed proteins in SF cell lysates according to the presence of MSU microcrystals under the polarized microscope. The shift to the right favors MSU [n=3], while a shift to the left favors the absence of MSU crystals (CPP or no crystals) [n=57]. Fold change refers to the difference in mean log2-normalised protein expression (NPX) values between samples of each category. See **Supplementary Table 1** for an explanation of the acronyms.

The comparison between MSU versus CPP or no crystals yielded notable results. Pairwise comparisons showed upregulation of several proteins in MSU-containing samples (**Supplementary Figures 2**, **S3**), most of which were confirmed in the volcano plots. Upregulation was particularly notable for ADA, OSM, CXCL1, CXCL6, CCL3, CCL4, CCL20, MCP3, TNFSF14, and IL-8 (**Figure 3**; supporting data in **Supplementary Table 4**). Similar results were observed when MSU was compared separately with CPP or no crystals (**Supplementary Figure 4**). The comparison between CPP versus no-crystal samples also showed some upregulation (such as uPA, HGF, CASP8, CCL3, and ST1A1), but to a lesser extent than for MSU (**Supplementary Figure 5**).

### 3.3 CXCL1, a candidate predictor of the presence of microcrystals

**Figure 4:**
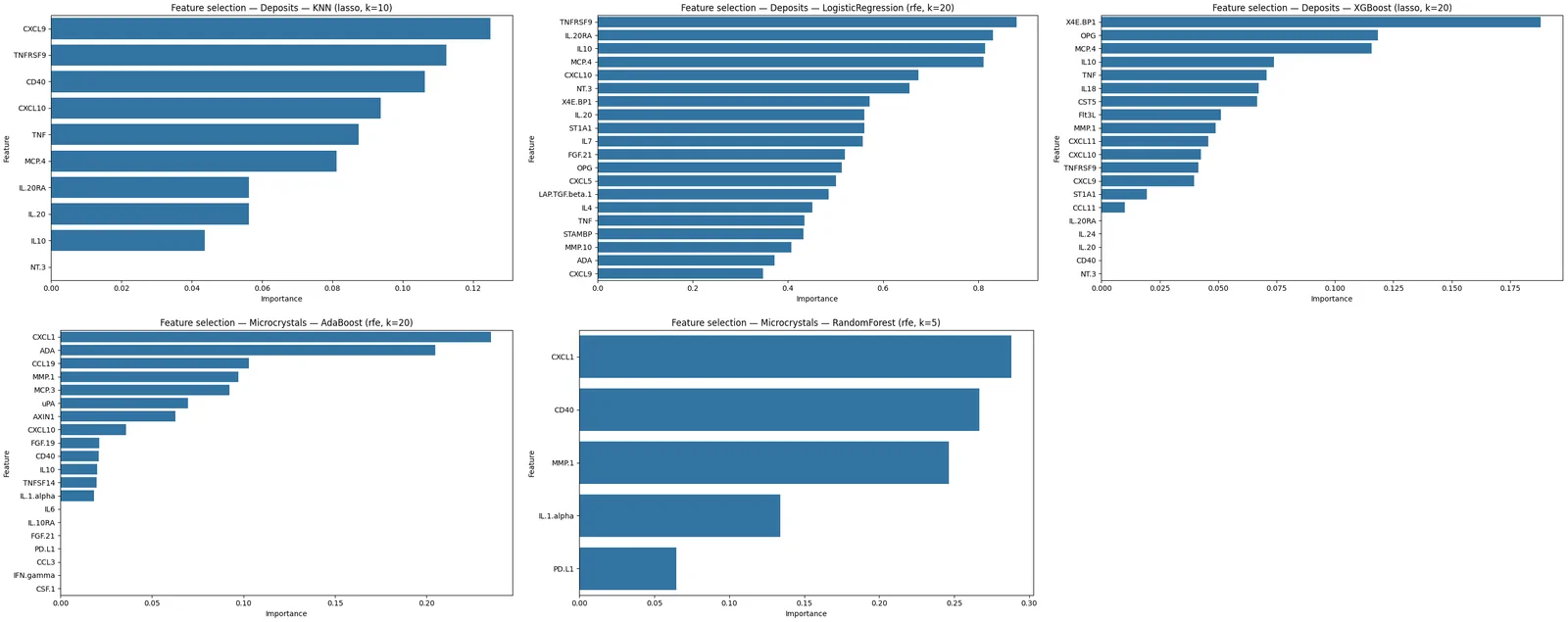
Classification models with selected features (proteins) and their contribution to each model. In row A, classification models for deposits. In row B, for microcrystals. See **Supplementary Table 1** for an explanation of the acronyms.

**Figure 4** shows the proteins contributing to classification models between groups.

For deposits, the best-performing models reached only moderate agreement, and the selected proteins varied notably (CXCL9 and TNFRSF9 in the KNN, TNFRSF9 and IL20RA in the LR, and X4E.BP1 and OPG in XGBoost). Their results are therefore of limited utility.

In the classification of microcrystals, CXCL1 was a leading contributor in both models. Other relevant proteins were ADA, CCL19, MMP1, CD40, or uPA. Notably, these proteins were already identified as differentially upregulated in MSU samples by the volcano plot.

Leukocytes were not significant contributors in any classification model.

### 3.4 Direct correlation between synovial fluid and serum found for chemokines and related proteins

**Figure 5:**
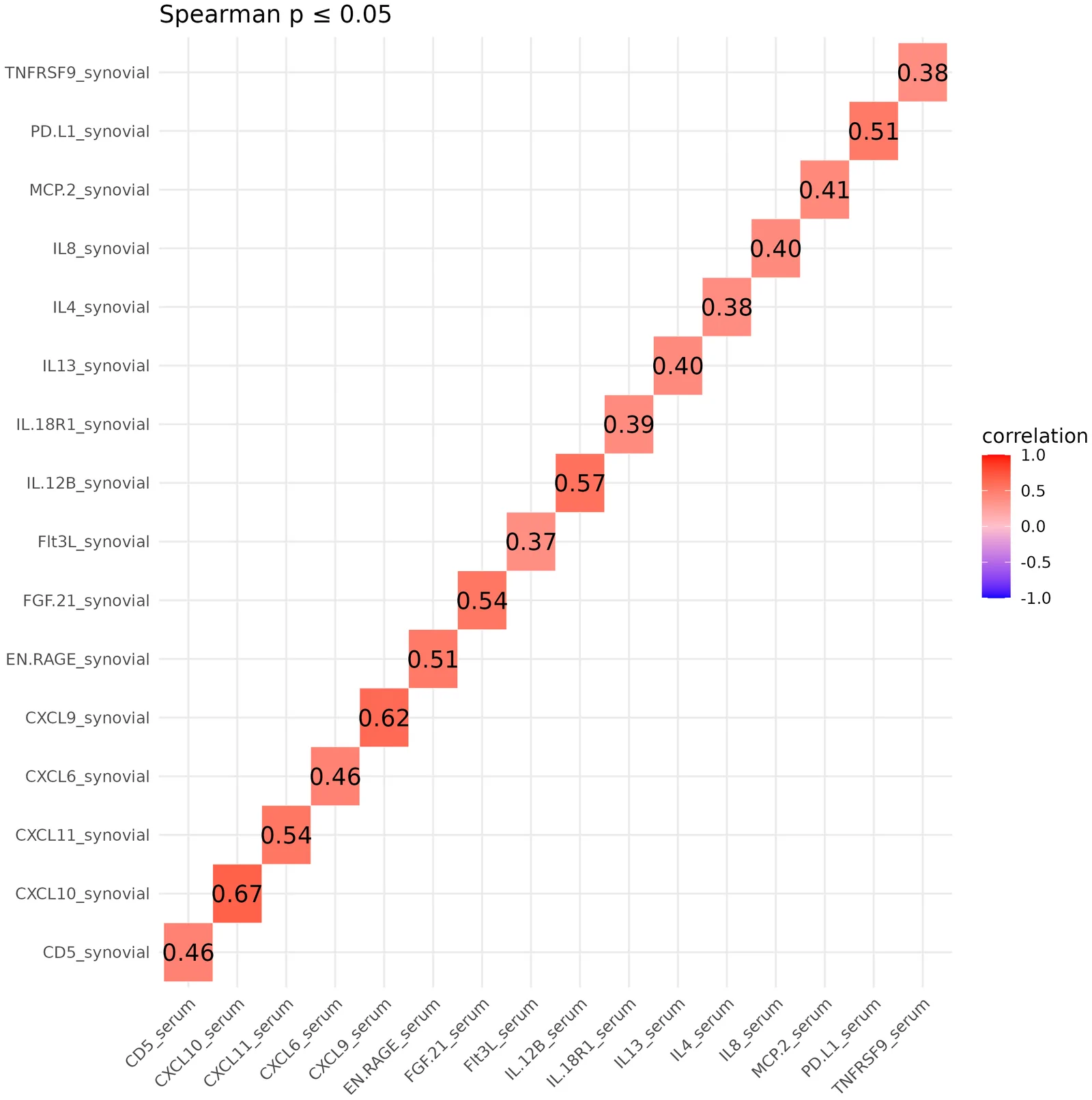
Heatmap showing proteins with a statistically significant (*P* < 0.050) correlation in their expression between paired synovial fluid and serum samples (n=28). The data depicted in the boxes are the ρ coefficient for each bivariate correlation. Red and blue boxes indicate a positive and negative correlation, respectively; the colour intensity indicates the strength of the correlation. Please refer to **Supplementary Table 1** for the explanation of acronyms.

When we compared SF to paired sera (n=28; **Figure 5**), a significant and positive correlation was observed for CXCL10, CXCL9, IL12B, FGF21, CXCL11, PDL1, EN-RAGE, CXCL6, CD5, MCP2, IL-13, IL-8, IL-18R1, IL-4, TNFRSF9, and FLT3L. No significant results were found for other proteins (full data in the **Supplementary Figure 6**).

## 4 Discussion

Our work confirms MSU crystal-related subclinical inflammation in SF is marked by increased leukocyte counts and a unique inflammatory proteome in SF cells. Proteins mainly relate to innate immunity and chemokine systems, with some showing serum upregulation. SF inflammation was unlinked to ultrasound deposits, perhaps most were located in the periarticular tissue or outside the joint cavity. Nevertheless, it highlights the importance of PLM for detecting crystal deposits and ongoing inflammation. MSU crystals are central to gout, causing flares and chronic low-grade inflammation that can damage joints and tissues. Understanding MSU-driven inflammation could guide future prevention and treatment strategies.

Our data support prior studies on gout and AH, showing high leukocyte counts with MSU crystals (16, 25). The numbers are consistent. Due to lab limitations, we could not characterize leukocytes further. Pascual reported mononuclear cells predominate in MSU samples (10.5% PMN) or crystal-free samples (0.4% of cells) (16). Leukocytes were lower in samples with CPP compared to MSU crystals. Martinez and Pascual previously noted increased cell counts, mainly mononuclear, in SF from CPP crystal disease (35). We enrolled asymptomatic participants based on hyperuricemia, so CPP crystals might be incidental. Conversely, in the Martinez and Pascual study, participants had symptomatic CPP disease with a subclinical inflammatory profile (36). We observed modest upregulation of proteins like uPA, CCL3, CASP-8, and ST1A1 in our samples. Further single-cell analysis of SF leukocytes, especially those in contact with MSU crystals, is needed. Until then, our proteomic cell lysate approach may be informative.

Proteins were notably upregulated in MSU samples, including TNFSF14 (37), ADA (38), and the OSM gene in THP-1 cells post-MSU exposure (39). A small study previously found IL6 and IL8 increased in gouty SF versus osteoarthritis (40). Since participants were asymptomatic, protein levels were likely lower than during flares, but further comparison is warranted. Chemokines CXCL1, CXCL6, and IL8, which attract neutrophils via CXCR2, were elevated, indicating their role in gout inflammation (41) and NETs formation (42), contributing to tophi (43) and bone erosion (44). Proteins like IL6 (37), TNFα (45), and IL1 isoforms (46), crucial for gout flares, showed negligible differences, implying the NLRP3-caspase1-IL1β pathway is inactive during intercritical periods. No change was observed in anti-inflammatory proteins such as TGFβ, despite prior reports (12), possibly indicating stabilization post-flare. Further research into the mechanisms of subclinical inflammation is needed.

Most protein expression showed no correlation between SF and serum, except for certain chemokines (CXCL10, CXCL9, CXCL11, CXCL6, IL8). Notably, chemokines are involved in insulin resistance, metabolic syndrome, hypertension, diabetes, and cardiovascular disease (47). Many of these bind to CXCR3 on vascular and endothelial cells (48). EN-RAGE (S100A12), an advanced glycation end-product binder linked to a 1.3-fold increase in the risk of cardiovascular diseases (49), is upregulated in MSU crystal-containing SF and shows parallel serum expression. Proteins related to cellular immunity (IL12B, PDL1, CD5, TNFRSF9) are worth further study; TNFSFR9 and, to a lesser extent, CD5 are upregulated in MSU-containing SF. Gout is an auto-inflammatory disease driven by innate immunity, with immune disturbances such as pro-inflammatory T cell shifts (50), imbalanced Th1/Th2, Th17/Treg, MAIT, and cytotoxic T-cell populations (51), low naïve CD8+ T cells, and increased memory and senescent CD8+ T cells (14). Our findings may clarify how hyperuricemia and MSU deposits affect cellular immunity.

Subclinical inflammation in AH and gout resembles other inflammasopathies like familial Mediterranean fever (52), cryopyrin-associated periodic syndrome (CAPS) (53), and atherosclerosis (54). Such low-grade inflammation can cause cardiovascular events (55) or amyloidosis and renal damage (52). In atherosclerosis, cholesterol crystals, lipid-laden cells, and poor clearance of dead cells lead to persistent inflammation (56). Similarly, ongoing MSU crystals appear to drive chronic inflammation in AH and gout. In CAPS, NLRP3-mutant monocytes produce IL18 but not IL1β, with a pro-inflammatory metabolic profile (53). Our data show IL18 and its receptor modestly upregulated in MSU samples. Future research should explore inflammasome activity in the presence of asymptomatic MSU deposits and the roles of soluble urate and MSU crystals as activators (57). Additional studies might examine epigenetic changes in SF cells related to hyperuricemia, which could explain the reduced gout flares under ULT before crystal removal (58).

Our study has several strengths. Two blinded observers simultaneously performed PLM immediately after aspiration. Additionally, a comprehensive proteomic assessment using Olink technology was chosen, as it enables studies with small sample volumes. Olink PEA was shown to be suitable for SF sample assessments, like serum or plasma (59). We also incorporated multivariate statistics and machine learning algorithms to achieve accurate differentiation among samples.

We acknowledge limitations, including performing proteomics on cell extracts due to infeasibility in SF solvents caused by volume and viscosity issues. Reducing viscosity with hyaluronidase might bias results, though studying cell lysates remains valuable and correlates well with SF and synovial cells (60). SF cells, mostly leukocytes, were identified by PLM, and not characterized or sorted. Correlation analyses between SF cell lysates and serum proteins showed significant results, especially for chemokines upregulated in the presence of crystals. MSU crystals were found in only five SF samples, possibly due to a periarticular location of the deposits or to a focus on joints with prominent effusion during ultrasound. Quality checks for crystals in proteomic samples were limited. Despite low prevalence, findings are valuable; future validation is needed. All participants were hyperuricemics, with high urate in SF, potentially contributing to inflammation. No joint aspiration in normouricemics for ethical reasons. Last flare timing in gout participants was not recorded, which may affect inflammation results. Similar findings in AH subjects suggest consistent effects. Future research should examine and compare the SF proteome during gout flares. Small sample sizes, especially for MSU, limit machine learning validation and require further external validation.

In summary, subclinical inflammation in SF linked to MSU crystals in people with non-symptomatic hyperuricemia features elevated leukocytes, active phagocytosis, and a distinct proteomic profile. Ultrasound-based crystal detection failed to show differences, supporting SF analysis as a means of detecting persistent inflammation. Some proteins helped differentiate SF microcrystals. Most inflammation is local to the joint, but serum chemokine activity suggests further study. The low prevalence of crystals in SF warrants further validation. Understanding this inflammatory profile may guide future control strategies in people with gout.

## Data Availability

The datasets presented in this study can be found in online repositories. The names of the repository/repositories and accession number(s) can be found below: Zenodo repository, ISABIAL community, at doi:10.5281/zenodo.8270344. The code used for statistical analyses and classification models is openly available at https://github.com/Samantao93/MSU-Synovitis-Omics.

## Supplementary material

Supplementary Table 1 List of proteins whose expression is assessed in the Olink Target 96 Inflammation panel.

Supplementary Table 2 Comparison between participants with asymptomatic hyperuricemia and intercritical gout and available synovial fluid assessment (n=81).

Supplementary Table 3 Volcano plot analyses for protein expression comparing groups of “Deposits”.

Supplementary Table 4 Volcano plot analyses for protein expression comparing groups of “Microcrystals”.

Supplementary Figure 1 Box plots showing the significant results in the bivariate comparison on the expression of IL-4 and MCP-2 in SF regarding the presence of ultrasound deposits (double-contour sign, tophus, and/or aggregate) at the aspirated joint. The box line represents the median; box limits are the first quartile (Q1, inferior) and third quartile (Q3, superior). The inferior whisker limit is Q1 minus 1.5xIQR, and the superior whisker limit is Q3 plus 1.5xIQR. Data points located below or above the whisker limits were considered outliers. IL4, interleukin-4; MCP2, monocyte chemotactic protein-2; NPX, normalized protein expression. **P* < 0.050.

Supplementary Figure 2 Box plots showing the bivariate comparisons of the relative protein expression in SF according to the presence and type of microcrystal by polarized light microscopy. Only significant comparisons with p<0.001 (Kruskal-Wallis test) are represented. The box line represents the median; box limits are the first quartile (Q1, inferior) and third quartile (Q3, superior). The inferior whisker limit is Q1 minus 1.5xIQR, and the superior whisker limit is Q3 plus 1.5xIQR. Data points located below or above the whisker limits were considered outliers. Please refer to **Supplementary Table 1** for an explanation of the abbreviations. *p<0.05. **p<0.01. ***p<0.001.

Supplementary Figure 3 Box plots showing the bivariate comparisons of the relative protein expression in SF according to the presence and type of microcrystal by polarized light microscopy. Only significant comparisons with p<0.05 but above p<0.001 (Kruskal-Wallis test) are represented. The box line represents the median; box limits are the first quartile (Q1, inferior) and third quartile (Q3, superior). The inferior whisker limit is Q1 minus 1.5xIQR, and the superior whisker limit is Q3 plus 1.5xIQR. Data points located below or above the whisker limits were considered outliers. Please refer to **Supplementary Table 1** for an explanation of the abbreviations. *p<0.05. **p<0.01. ***p<0.001.

Supplementary Figure 4 Volcano plot of differentially expressed proteins in SF samples according to the presence and type of microcrystals under polarized light microscopy. The shift to the right favors MSU, while a shift to the left favors no crystals (**Supplementary Figure 1A**) or CPP crystals (**Supplementary Figure 1B**). Fold change refers to the difference in mean log2-normalized protein expression (NPX) values between samples of each category. Please refer to **Supplementary Table 1** for an explanation of the abbreviations.

Supplementary Figure 5 Volcano plot of differentially expressed proteins in SF samples comparing the presence versus the absence of CPP crystals under the polarized light microscope. The shift to the right favors CPP, while a shift to the left favors no crystals. Fold change refers to the difference in mean log2-normalized protein expression (NPX) values between samples of each category. See **Figure 5** legend for an explanation of the abbreviations.

Supplementary Figure 6 Correlation in normalized protein expression between paired synovial fluid and serum samples in 28 participants. The depicted data in boxes is the ρ coefficient for each bivariate correlation. Red and blue boxes indicate a positive and negative correlation, respectively; the color intensity is related to the strength of the correlation. Please refer to **Supplementary Table 1** for the explanation of the abbreviations.

Supplementary Annex I Machine learning classification models – Methods. As classification models, we implemented a diverse set of algorithms: logistic regression (LR), random forest (RF), XGBoost, support vector machine (SVM), naive Bayes, K-nearest neighbors (KNN), LightGBM, AdaBoost, and CatBoost. The following feature selection strategies were applied to optimize model performance: all features (no feature reduction, retaining all original features), ANOVA F-test (f_classif), chi-2 test, recursive feature elimination (RFE), lasso regularization, and ridge regularization. Each method was tested with multiple values of k, representing the number of top-ranked features selected. All models were trained using default parameters, with modifications applied where necessary for compatibility. Model training was performed using the features selected by each method and repeated across varying feature counts (5, 10, 15, and 20). Models’ performance was evaluated on the test set using standard classification metrics, such as accuracy, precision, recall (sensitivity), F1-score (harmonic mean of precision and recall), AUC (area under the receiver operating characteristic curve), and Cohen’s kappa (to provide a more precise assessment given the class imbalance in the dataset). The complete evaluation of model classification is provided in the Spreadsheets 1-2. Accordingly, we selected the following models: For *deposits*, KNN with lasso-10 (kappa 0.67, AUC 0.65, accuracy 0.88), LR with RFE-20 (kappa 0.54, AUC 0.73, accuracy 0.81), and XGBoost with lasso-20 (kappa 0.59, AUC 0.80, accuracy 0.81). For *microcrystals*, models were AdaBoost with RFE-20 (kappa 0.78, AUC 0.91, accuracy 0.94) and RF with RFE-5 (kappa 0.78, AUC 0.91, accuracy 0.88).
