# Supplementary material for: Subclinical inflammation associated with monosodium urate crystal deposition: a cellular and proteomic study in synovial fluid
Source: Front Immunol. 2026 Aug 6;17:1888214. doi: 10.3389/fimmu.2026.1888214 (PMC13490104; doi:10.3389/fimmu.2026.1888214)
Supplement: Supplementary file 1 [file Table1.docx]

**Subclinical Inflammation Associated with Monosodium Urate Crystal Deposition: A Cellular and Proteomic Study in Synovial Fluid.**

**Supplementary Tables**

**Table S1**. **List of proteins whose expression is assessed in the Olink Target 96 Inflammation panel.**

| **Abbreviation** | **Full name** |
| --- | --- |
| ADA | Adenosine deaminase |
| ARTN | Artemin |
| AXIN1 | Axin-1 |
| CASP8 | Caspase-8 |
| CCL11 | Eotaxin |
| CCL13 | C-C motif chemokine 13 |
| CCL19 | C-C motif chemokine 19 |
| CCL2 | C-C motif chemokine 2 |
| CCL20 | C-C motif chemokine 20 |
| CCL23 | C-C motif chemokine 23 |
| CCL25 | C-C motif chemokine 25 |
| CCL28 | C-C motif chemokine 28 |
| CCL3 | C-C motif chemokine 3 |
| CCL4 | C-C motif chemokine 4 |
| CCL7 | C-C motif chemokine 7 (also known as monocyte-chemotactic protein 3 -MCP3) |
| CCL8 | C-C motif chemokine 8 (also known as monocyte-chemotactic protein 2 -MCP2) |
| CD244 | Natural killer cell receptor 2B4 |
| CD274 | Programmed cell death 1 ligand 1 (or PD-L1) |
| CD40 | Tumor necrosis factor receptor superfamily member 5 |
| CD5 | T-cell surface glycoprotein CD5 |
| CD6 | T-cell differentiation antigen CD6 |
| CD8A | T-cell surface glycoprotein CD8 alpha chain |
| CDCP1 | CUB domain-containing protein 1 |
| CSF1 | Macrophage colony-stimulating factor 1 |
| CST5 | Cystatin-D |
| CX3CL1 | Fractalkine |
| CXCL1 | Growth-regulated alpha protein |
| CXCL10 | C-X-C motif chemokine 10 |
| CXCL11 | C-X-C motif chemokine 11 |
| CXCL5 | C-X-C motif chemokine 5 |
| CXCL6 | C-X-C motif chemokine 6 |
| CXCL9 | C-X-C motif chemokine 9 |
| DNER | Delta and Notch-like epidermal growth factor-related receptor |
| EIF4EBP1 | Eukaryotic translation initiation factor 4E-binding protein 1 |
| FGF19 | Fibroblast growth factor 19 |
| FGF21 | Fibroblast growth factor 21 |
| FGF23 | Fibroblast growth factor 23 |
| FGF5 | Fibroblast growth factor 5 |
| FLT3L | Fms-related tyrosine kinase 3 ligand |
| GDNF | Glial cell line-derived neurotrophic factor |
| HGF | Hepatocyte growth factor |
| IFNG | Interferon gamma |
| IL10 | Interleukin-10 |
| IL10RA | Interleukin-10 receptor subunit alpha |
| IL10RB | Interleukin-10 receptor subunit beta |
| IL12B | Interleukin-12 subunit beta |
| IL13 | Interleukin-13 |
| IL15RA | Interleukin-15 receptor subunit alpha |
| IL17A | Interleukin-17A |
| IL17C | Interleukin-17C |
| IL18 | Interleukin-18 |
| IL18R1 | Interleukin-18 receptor 1 |
| IL1A | Interleukin-1 alpha |
| IL2 | Interleukin-2 |
| IL20 | Interleukin-20 |
| IL20RA | Interleukin-20 receptor subunit alpha |
| IL22RA1 | Interleukin-22 receptor subunit alpha-1 |
| IL24 | Interleukin-24 |
| IL2RB | Interleukin-2 receptor subunit beta |
| IL33 | Interleukin-33 |
| IL4 | Interleukin-4 |
| IL5 | Interleukin-5 |
| IL6 | Interleukin-6 |
| IL7 | Interleukin-7 |
| IL8 | Interleukin-8 (also known as C-X-C motif chemokine 8 or CXCL8) |
| KITLG | Kit ligand |
| LIF | Leukemia inhibitory factor |
| LIFR | Leukemia inhibitory factor receptor |
| LTA | Lymphotoxin-alpha |
| MMP1 | Interstitial collagenase |
| MMP10 | Stromelysin-2 |
| NGF | Beta-nerve growth factor |
| NRTN | Neurturin |
| NTF3 | Neurotrophin-3 |
| OSM | Oncostatin-M |
| S100A12 | Protein S100-A12 (also kwon as EN-RAGE) |
| SIRT2 | NAD-dependent protein deacetylase sirtuin-2 |
| SLAMF1 | Signaling lymphocytic activation molecule |
| STAMBP | STAM-binding protein |
| ST1A1 | Sulfotransferase 1A1 |
| TGFA | Protransforming growth factor alpha |
| TGFB1 | Transforming growth factor beta-1 proprotein |
| TNF | Tumor necrosis factor |
| TNFRSF11B | Tumor necrosis factor receptor superfamily member 11B |
| TNFRSF9 | Tumor necrosis factor receptor superfamily member 9 |
| TNFSF10 | Tumor necrosis factor ligand superfamily member 10 (also known as TRAIL) |
| TNFSF11 | Tumor necrosis factor ligand superfamily member 11 (also known as TRANCE or RANK-ligand) |
| TNFSF12 | Tumor necrosis factor ligand superfamily member 12 |
| TNFSF14 | Tumor necrosis factor ligand superfamily member 14 |
| TSLP | Thymic stromal lymphopoietin |
| uPA | Urokinase-type plasminogen activator |
| VEGFA | Vascular endothelial growth factor A |
