# Supplementary material for: Subclinical inflammation associated with monosodium urate crystal deposition: a cellular and proteomic study in synovial fluid
Source: Front Immunol. 2026 Aug 6;17:1888214. doi: 10.3389/fimmu.2026.1888214 (PMC13490104; doi:10.3389/fimmu.2026.1888214)
Supplement: Supplementary file 2 [file Table2.docx]

**Subclinical Inflammation Associated with Monosodium Urate Crystal Deposition: A Cellular and Proteomic Study in Synovial Fluid.**

**Supplementary Tables**

**Table S2. Comparison between participants with asymptomatic hyperuricemia and intercritical gout and available synovial fluid assessment (n=81).**

| Variables | AH (n=62) | Gout (n=19) | *P* |
| --- | --- | --- | --- |
| Demographics and clinical data |  |  |  |
| Age, in years, median (IQR) | 66.0 (51.0-77.0) | 61.0 (55.0-80.0) | 0.884 |
| Men | 44 (68.8) | 15 78.9) | 0.389 |
| White | 56 (87.5) | 19 (100) | 0.188 |
| Comorbidity index, median (IQR) | 4.0 (2.0-5.0) | 3.0 (1.0-5.0) | 0.209 |
| Hypertension | 47 (73.4) | 12 (63.2) | 0.385 |
| Diabetes mellitus | 17 (26.6) | 3 (15.8) | 0.542 |
| Dyslipidemia | 46 (71.9) | 15 (78.9) | 0.540 |
| Smoking background | 35 (54.7) | 14 (73.7) | 0.139 |
| Established cardiovascular disease | 25 (39.1) | 3 (15.8) | 0.060 |
| Body mass index, in kg/m^2^, median (IQR) | 30.6 (27.1-33.8) | 30.2 (28.5-34.6) | 0.854 |
| Laboratory data |  |  |  |
| Urate, in mg/dL, median (IQR) | 7.4 (6.9-8.4) | 7.7 (6.9-8.7) | 0.475 |
| eGFR, in mL/min/1.73m^2^, median (IQR) | 73.2 (51.4-90.9) | 80.7 (56.1-100.8) | 0.325 |
| hsCRP, in mg/dL, median (IQR) | 0.2 (0.1-0.7) | 0.3 (0.2-0.8) | 0.294 |
| ESR, in mm/h, median (IQR) [n=78] | 22.0 (10.0-33.0) | 18.0 (11.0-49.0) | 0.792 |
| SAA, in ng/mL, median (IQR) [n=80] | 5.0 (4.0-10.0) | 7.0 (4.0-12.0) | 0.682 |
| Total leukocytes, per mm^3^, median (IQR) | 7830.0 (6590.0-9540.0) | 8770.0 (6760.0-10140.0) | 0.549 |
| Neutrophil:lymphocyte ratio, median (IQR) | 2.4 (1.7-3.0) | 2.4 (1.8-3.7) | 0.342 |

***IQR****: interquartile range.* ***AH****: asymptomatic hyperuricemia;* ***eGFR****: estimated glomerular filtration rate, by CKD-EPI equation;* ***hsCRP****: high-sensitivity C-reactive protein;* ***ESR****: erythrocyte sedimentation rate;* ***SAA****: serum amyloid A;* ***MTP****: metatarsophalangeal.*

*ªData presented as n(%) unless stated otherwise.*
