# Supplementary material for: Subclinical inflammation associated with monosodium urate crystal deposition: a cellular and proteomic study in synovial fluid
Source: Front Immunol. 2026 Aug 6;17:1888214. doi: 10.3389/fimmu.2026.1888214 (PMC13490104; doi:10.3389/fimmu.2026.1888214)
Supplement: Supplementary file 3 [file Table3.docx]

**Subclinical Inflammation Associated with Monosodium Urate Crystal Deposition: A Cellular and Proteomic Study in Synovial Fluid.**

**Supplementary Tables**

**Table S3. Volcano plot analyses for protein expression comparing groups of “Deposits”.**

| **Protein** | **logFC** | **AveExpr** | **t** | ***P*** | **Adjusted *P*** | **B** | **minus_log10_*P*** |
| --- | --- | --- | --- | --- | --- | --- | --- |
| *IL.20* | 0.072 | 0.025 | 2.041 | 0.046 | 0.748 | -3.791 | 1.334 |
| *MCP.3* | 0.606 | 1.306 | 2.034 | 0.047 | 0.748 | -3.802 | 1.327 |
| *EN.RAGE* | 0.782 | 0.807 | 1.935 | 0.058 | 0.748 | -3.953 | 1.233 |
| *IL10* | -0.154 | 0.323 | -1.928 | 0.059 | 0.748 | -3.963 | 1.227 |
| *SCF* | -0.354 | 1.54 | -1.894 | 0.064 | 0.748 | -4.013 | 1.195 |
| *MMP.10* | -0.727 | 3.191 | -1.848 | 0.07 | 0.748 | -4.079 | 1.153 |
| *CCL28* | -0.158 | 0.318 | -1.803 | 0.077 | 0.748 | -4.144 | 1.112 |
| *SIRT2* | 0.454 | 0.877 | 1.791 | 0.079 | 0.748 | -4.161 | 1.101 |
| *LIF* | 0.173 | 0.184 | 1.663 | 0.102 | 0.748 | -4.334 | 0.99 |
| *IL4* | -0.033 | 0.235 | -1.648 | 0.105 | 0.748 | -4.353 | 0.977 |
| *OSM* | 0.477 | 0.867 | 1.6 | 0.116 | 0.748 | -4.414 | 0.937 |
| *FGF.19* | -0.342 | 0.963 | -1.582 | 0.12 | 0.748 | -4.438 | 0.922 |
| *IL7* | 0.022 | 0.007 | 1.561 | 0.125 | 0.748 | -4.464 | 0.905 |
| *NT.3* | 0.025 | 0.787 | 1.559 | 0.125 | 0.748 | -4.466 | 0.903 |
| *Flt3L* | -0.502 | 4.203 | -1.557 | 0.126 | 0.748 | -4.469 | 0.901 |
| *CXCL6* | 0.471 | 1.591 | 1.487 | 0.143 | 0.748 | -4.553 | 0.844 |
| *ADA* | 0.692 | 0.869 | 1.448 | 0.154 | 0.748 | -4.599 | 0.813 |
| *MCP.4* | -0.448 | 7.516 | -1.425 | 0.16 | 0.748 | -4.626 | 0.795 |
| *CXCL1* | 0.716 | 4.251 | 1.397 | 0.168 | 0.748 | -4.657 | 0.773 |
| *FGF.21* | -0.302 | 0.545 | -1.388 | 0.171 | 0.748 | -4.668 | 0.766 |
| *CCL20* | 0.339 | 1.31 | 1.363 | 0.179 | 0.748 | -4.695 | 0.747 |
| *CST5* | -0.289 | 1.26 | -1.287 | 0.204 | 0.748 | -4.776 | 0.691 |
| *IL.24* | 0.045 | 0.236 | 1.238 | 0.221 | 0.748 | -4.826 | 0.655 |
| *CCL23* | -0.356 | 3.153 | -1.238 | 0.221 | 0.748 | -4.826 | 0.655 |
| *STAMBP* | 0.607 | 1.693 | 1.209 | 0.232 | 0.748 | -4.855 | 0.634 |
| *IL13* | 0.024 | 0.393 | 1.168 | 0.248 | 0.748 | -4.894 | 0.606 |
| *TNFSF14* | 0.547 | 0.911 | 1.134 | 0.262 | 0.748 | -4.926 | 0.582 |
| *SLAMF1* | -0.06 | 0.586 | -1.125 | 0.266 | 0.748 | -4.935 | 0.575 |
| *X4E.BP1* | 0.384 | 0.498 | 1.123 | 0.266 | 0.748 | -4.936 | 0.574 |
| *OPG* | -0.369 | 7.01 | -1.103 | 0.275 | 0.748 | -4.954 | 0.56 |
| *IL8* | 0.798 | 3.624 | 1.094 | 0.279 | 0.748 | -4.962 | 0.554 |
| *TNF* | 0.145 | 0.267 | 1.092 | 0.28 | 0.748 | -4.964 | 0.553 |
| *IL.22.RA1* | -0.014 | 2.174 | -1.074 | 0.288 | 0.748 | -4.98 | 0.541 |
| *CCL11* | -0.141 | 0.278 | -1.059 | 0.294 | 0.748 | -4.993 | 0.531 |
| *FGF.23* | -0.065 | 0.916 | -1.041 | 0.303 | 0.748 | -5.008 | 0.519 |
| *CD6* | 0.237 | 0.471 | 1.037 | 0.304 | 0.748 | -5.012 | 0.516 |
| *TRANCE* | 0.263 | 0.56 | 1.025 | 0.31 | 0.748 | -5.022 | 0.509 |
| *IL.1.alpha* | 0.297 | 1.972 | 0.994 | 0.325 | 0.748 | -5.047 | 0.488 |
| *CXCL9* | -0.322 | 2.012 | -0.985 | 0.329 | 0.748 | -5.055 | 0.483 |
| *TNFRSF9* | 0.223 | 1.408 | 0.951 | 0.346 | 0.748 | -5.082 | 0.461 |
| *CASP.8* | 0.553 | 3.319 | 0.934 | 0.355 | 0.748 | -5.095 | 0.45 |
| *TRAIL* | 0.152 | 0.616 | 0.93 | 0.357 | 0.748 | -5.098 | 0.448 |
| *CDCP1* | 0.295 | 0.63 | 0.926 | 0.359 | 0.748 | -5.101 | 0.445 |
| *IL.2RB* | 0.019 | 1.245 | 0.92 | 0.362 | 0.748 | -5.106 | 0.441 |
| *CCL3* | 0.656 | 2.364 | 0.896 | 0.374 | 0.757 | -5.123 | 0.427 |
| *CXCL10* | -0.323 | 3.765 | -0.875 | 0.385 | 0.763 | -5.138 | 0.414 |
| *CCL19* | -0.238 | 3.972 | -0.845 | 0.402 | 0.763 | -5.16 | 0.396 |
| *IL33* | 0.045 | 0.198 | 0.84 | 0.405 | 0.763 | -5.164 | 0.392 |
| *IL.12B* | -0.075 | 0.233 | -0.83 | 0.41 | 0.763 | -5.17 | 0.387 |
| *PD.L1* | 0.099 | 1.681 | 0.808 | 0.423 | 0.771 | -5.185 | 0.374 |
| *IL.18R1* | 0.204 | 1.401 | 0.788 | 0.434 | 0.776 | -5.198 | 0.362 |
| *IL.20RA* | 0.045 | 0.602 | 0.761 | 0.45 | 0.79 | -5.216 | 0.347 |
| *DNER* | -0.031 | 0.05 | -0.721 | 0.474 | 0.801 | -5.24 | 0.324 |
| *AXIN1* | 0.081 | 0.151 | 0.721 | 0.474 | 0.801 | -5.24 | 0.324 |
| *CCL25* | -0.023 | 0.015 | -0.708 | 0.482 | 0.801 | -5.248 | 0.317 |
| *IL.17C* | -0.016 | 0.38 | -0.644 | 0.523 | 0.838 | -5.284 | 0.282 |
| *CXCL5* | 0.159 | 1.48 | 0.633 | 0.53 | 0.838 | -5.29 | 0.276 |
| *NRTN* | -0.006 | 0.004 | -0.63 | 0.532 | 0.838 | -5.291 | 0.274 |
| *FGF.5* | 0.015 | 0.017 | 0.602 | 0.55 | 0.843 | -5.305 | 0.26 |
| *MCP.2* | -0.169 | 2.803 | -0.597 | 0.553 | 0.843 | -5.308 | 0.257 |
| *IL.15RA* | -0.019 | 0.03 | -0.579 | 0.565 | 0.847 | -5.317 | 0.248 |
| *VEGFA* | -0.131 | 5.121 | -0.537 | 0.594 | 0.876 | -5.336 | 0.226 |
| *IFN.gamma* | 0.146 | 2.089 | 0.516 | 0.608 | 0.876 | -5.345 | 0.216 |
| *TGF.alpha* | -0.004 | 0.003 | -0.504 | 0.616 | 0.876 | -5.35 | 0.21 |
| *CD5* | 0.273 | 1.097 | 0.495 | 0.623 | 0.876 | -5.354 | 0.206 |
| *IL.10RB* | 0.056 | 0.228 | 0.483 | 0.631 | 0.876 | -5.359 | 0.2 |
| *IL2* | -0.032 | 0.552 | -0.456 | 0.65 | 0.888 | -5.37 | 0.187 |
| *LAP.TGF.beta.1* | 0.146 | 1.5 | 0.444 | 0.659 | 0.888 | -5.374 | 0.181 |
| *CX3CL1* | -0.003 | 0.627 | -0.414 | 0.68 | 0.903 | -5.385 | 0.167 |
| *CCL4* | 0.231 | 1.709 | 0.402 | 0.689 | 0.903 | -5.389 | 0.161 |
| *uPA* | 0.139 | 5.135 | 0.335 | 0.739 | 0.944 | -5.41 | 0.131 |
| *TWEAK* | -0.118 | 3.251 | -0.332 | 0.741 | 0.944 | -5.41 | 0.13 |
| *HGF* | 0.123 | 3.909 | 0.318 | 0.751 | 0.944 | -5.414 | 0.124 |
| *IL6* | -0.095 | 0.921 | -0.284 | 0.778 | 0.954 | -5.423 | 0.109 |
| *IL.10RA* | 0.002 | 0.001 | 0.282 | 0.779 | 0.954 | -5.423 | 0.108 |
| *CD244* | 0.08 | 2.026 | 0.234 | 0.816 | 0.985 | -5.434 | 0.088 |
| *MCP.1* | -0.075 | 6.059 | -0.174 | 0.862 | 0.995 | -5.444 | 0.064 |
| *CD8A* | -0.014 | 0.063 | -0.149 | 0.882 | 0.995 | -5.447 | 0.054 |
| *CSF.1* | 0.049 | 3.637 | 0.145 | 0.885 | 0.995 | -5.448 | 0.053 |
| *TSLP* | 0.004 | 1 | 0.143 | 0.887 | 0.995 | -5.448 | 0.052 |
| *IL.17A* | 0.006 | 0.04 | 0.141 | 0.888 | 0.995 | -5.448 | 0.051 |
| *IL18* | 0.056 | 2.594 | 0.138 | 0.89 | 0.995 | -5.448 | 0.05 |
| *GDNF* | 0.005 | 1.356 | 0.114 | 0.91 | 0.995 | -5.451 | 0.041 |
| *ARTN* | -0.006 | 0.486 | -0.094 | 0.925 | 0.995 | -5.453 | 0.034 |
| *MMP.1* | 0.057 | 6.875 | 0.087 | 0.931 | 0.995 | -5.453 | 0.031 |
| *ST1A1* | -0.052 | 4.944 | -0.071 | 0.944 | 0.995 | -5.454 | 0.025 |
| *TNFB* | -0.004 | 0.394 | -0.07 | 0.944 | 0.995 | -5.454 | 0.025 |
| *CD40* | 0.032 | 6.973 | 0.06 | 0.952 | 0.995 | -5.455 | 0.021 |
| *IL5* | 0.002 | 0.468 | 0.02 | 0.984 | 1 | -5.456 | 0.007 |
| *CXCL11* | 0.005 | 0.532 | 0.017 | 0.986 | 1 | -5.456 | 0.006 |
| *LIF.R* | 0 | 0 | 0 | 1 | 1 | -5.457 | 0 |
| *Beta.NGF* | 0 | 0.278 | 0 | 1 | 1 | -5.457 | 0 |

*For abbreviation explanation, please refer to Table S1 of the Supplementary Material.*
