# Supplementary material for: Subclinical inflammation associated with monosodium urate crystal deposition: a cellular and proteomic study in synovial fluid
Source: Front Immunol. 2026 Aug 6;17:1888214. doi: 10.3389/fimmu.2026.1888214 (PMC13490104; doi:10.3389/fimmu.2026.1888214)
Supplement: Supplementary file 4 [file Table4.docx]

**Subclinical Inflammation Associated with Monosodium Urate Crystal Deposition: A Cellular and Proteomic Study in Synovial Fluid.**

**Supplementary Tables**

**Table S4. Volcano plot analyses for protein expression comparing groups of “Microcrystals”.**

| **Protein** | **logFC** | **AveExpr** | **t** | ***P*** | **Adjusted *P*** | **B** | **minus_log10_*P*** |
| --- | --- | --- | --- | --- | --- | --- | --- |
| *MCP.3* | 3.998 | 1.265 | 13.793 | <0.001 | <0.001 | 35.291 | 19.346 |
| *CCL20* | 3.197 | 1.265 | 12.338 | <0.001 | <0.001 | 30.389 | 17.221 |
| *OSM* | 3.723 | 0.804 | 10.808 | <0.001 | <0.001 | 24.946 | 14.861 |
| *ADA* | 5.793 | 0.774 | 10.123 | <0.001 | <0.001 | 22.419 | 13.766 |
| *CXCL1* | 6.194 | 4.131 | 9.585 | <0.001 | <0.001 | 20.400 | 12.889 |
| *LIF* | 1.185 | 0.171 | 8.694 | <0.001 | <0.001 | 17.002 | 11.413 |
| *CXCL6* | 3.646 | 1.516 | 8.622 | <0.001 | <0.001 | 16.728 | 11.294 |
| *TNFRSF9* | 2.583 | 1.340 | 7.836 | <0.001 | <0.001 | 13.689 | 9.971 |
| *SIRT2* | 2.782 | 0.819 | 7.739 | <0.001 | <0.001 | 13.313 | 9.807 |
| *IL8* | 7.826 | 3.528 | 7.643 | <0.001 | <0.001 | 12.938 | 9.644 |
| *TNFSF14* | 5.104 | 0.835 | 7.517 | <0.001 | <0.001 | 12.453 | 9.432 |
| *EN.RAGE* | 4.376 | 0.730 | 7.514 | <0.001 | <0.001 | 12.441 | 9.427 |
| *CDCP1* | 3.339 | 0.603 | 7.513 | <0.001 | <0.001 | 12.437 | 9.425 |
| *TRAIL* | 1.708 | 0.575 | 7.269 | <0.001 | <0.001 | 11.489 | 9.012 |
| *CCL3* | 7.342 | 2.335 | 6.719 | <0.001 | <0.001 | 9.367 | 8.084 |
| *IL.18R1* | 2.536 | 1.358 | 6.573 | <0.001 | <0.001 | 8.806 | 7.839 |
| *CD6* | 2.232 | 0.428 | 6.544 | <0.001 | <0.001 | 8.696 | 7.790 |
| *TNF* | 1.302 | 0.242 | 6.538 | <0.001 | <0.001 | 8.672 | 7.780 |
| *CCL4* | 5.551 | 1.691 | 6.484 | <0.001 | <0.001 | 8.464 | 7.689 |
| *IL7* | 0.131 | 0.007 | 6.296 | <0.001 | <0.001 | 7.748 | 7.375 |
| *TRANCE* | 2.415 | 0.531 | 6.206 | <0.001 | <0.001 | 7.404 | 7.223 |
| *MCP.1* | 4.030 | 5.998 | 6.055 | <0.001 | <0.001 | 6.833 | 6.973 |
| *uPA* | 3.860 | 5.066 | 5.934 | <0.001 | <0.001 | 6.379 | 6.773 |
| *PD.L1* | 1.107 | 1.662 | 5.789 | <0.001 | <0.001 | 5.835 | 6.534 |
| *CASP.8* | 5.350 | 3.317 | 5.588 | <0.001 | <0.001 | 5.089 | 6.205 |
| *CD244* | 2.968 | 1.955 | 5.427 | <0.001 | <0.001 | 4.493 | 5.942 |
| *HGF* | 3.298 | 3.873 | 5.237 | <0.001 | <0.001 | 3.805 | 5.638 |
| *CXCL5* | 2.110 | 1.438 | 5.229 | <0.001 | <0.001 | 3.774 | 5.624 |
| *AXIN1* | 0.945 | 0.148 | 5.207 | <0.001 | <0.001 | 3.696 | 5.589 |
| *CD5* | 4.614 | 1.050 | 5.083 | <0.001 | <0.001 | 3.250 | 5.392 |
| *IL.24* | 0.301 | 0.233 | 5.073 | <0.001 | <0.001 | 3.212 | 5.375 |
| *IL18* | 3.386 | 2.589 | 5.013 | <0.001 | <0.001 | 3.000 | 5.280 |
| *CSF.1* | 2.815 | 3.640 | 4.962 | <0.001 | <0.001 | 2.817 | 5.199 |
| *TWEAK* | 2.877 | 3.243 | 4.739 | <0.001 | <0.001 | 2.035 | 4.851 |
| *STAMBP* | 3.934 | 1.656 | 4.601 | <0.001 | <0.001 | 1.559 | 4.639 |
| *IFN.gamma* | 2.065 | 2.049 | 4.348 | <0.001 | <0.001 | 0.704 | 4.256 |
| *LAP.TGF.beta.1* | 2.417 | 1.460 | 4.269 | <0.001 | <0.001 | 0.443 | 4.139 |
| *ST1A1* | 5.361 | 4.968 | 4.089 | <0.001 | <0.001 | -0.147 | 3.874 |
| *IL.10RB* | 0.807 | 0.201 | 3.979 | <0.001 | <0.001 | -0.498 | 3.715 |
| *CD40* | 3.320 | 6.909 | 3.391 | 0.001 | 0.003 | -2.280 | 2.903 |
| *CCL19* | 1.806 | 4.062 | 3.252 | 0.002 | 0.004 | -2.676 | 2.721 |
| *IL.20* | 0.209 | 0.022 | 3.220 | 0.002 | 0.005 | -2.764 | 2.680 |
| *CXCL9* | 1.775 | 1.958 | 2.891 | 0.005 | 0.012 | -3.645 | 2.269 |
| *MCP.2* | 1.529 | 2.766 | 2.868 | 0.006 | 0.012 | -3.701 | 2.242 |
| *MMP.1* | 3.602 | 6.815 | 2.826 | 0.006 | 0.013 | -3.809 | 2.192 |
| *VEGFA* | 1.187 | 5.104 | 2.631 | 0.011 | 0.022 | -4.288 | 1.964 |
| *MCP.4* | 1.456 | 7.471 | 2.364 | 0.021 | 0.042 | -4.899 | 1.669 |
| *IL.1.alpha* | 1.241 | 2.004 | 2.139 | 0.037 | 0.070 | -5.372 | 1.437 |
| *IL6* | 1.624 | 0.934 | 2.119 | 0.038 | 0.072 | -5.412 | 1.417 |
| *X4E.BP1* | 1.330 | 0.476 | 2.027 | 0.047 | 0.087 | -5.592 | 1.326 |
| *CXCL11* | 1.132 | 0.473 | 1.919 | 0.060 | 0.108 | -5.794 | 1.223 |
| *CXCL10* | 1.097 | 3.698 | 1.518 | 0.134 | 0.238 | -6.460 | 0.872 |
| *FGF.19* | -0.593 | 0.956 | -1.416 | 0.162 | 0.281 | -6.607 | 0.790 |
| *IL33* | 0.115 | 0.190 | 1.125 | 0.265 | 0.452 | -6.972 | 0.576 |
| *SCF* | -0.396 | 1.523 | -1.065 | 0.291 | 0.486 | -7.037 | 0.535 |
| *CCL23* | 0.588 | 3.135 | 1.052 | 0.297 | 0.486 | -7.051 | 0.527 |
| *IL.12B* | 0.194 | 0.227 | 1.043 | 0.301 | 0.486 | -7.060 | 0.521 |
| *IL.10RA* | 0.009 | 0.000 | 1.032 | 0.306 | 0.486 | -7.072 | 0.514 |
| *CD8A* | 0.178 | 0.056 | 1.014 | 0.315 | 0.491 | -7.089 | 0.502 |
| *Flt3L* | 0.614 | 4.165 | 0.969 | 0.337 | 0.516 | -7.134 | 0.473 |
| *TNFB* | 0.089 | 0.385 | 0.904 | 0.369 | 0.555 | -7.195 | 0.432 |
| *IL.20RA* | 0.104 | 0.590 | 0.896 | 0.374 | 0.555 | -7.203 | 0.427 |
| *IL5* | -0.160 | 0.456 | -0.855 | 0.396 | 0.579 | -7.239 | 0.402 |
| *IL2* | -0.113 | 0.539 | -0.836 | 0.406 | 0.584 | -7.254 | 0.391 |
| *MMP.10* | -0.653 | 3.205 | -0.813 | 0.419 | 0.594 | -7.273 | 0.377 |
| *FGF.23* | -0.090 | 0.908 | -0.742 | 0.461 | 0.643 | -7.329 | 0.336 |
| *ARTN* | -0.088 | 0.477 | -0.712 | 0.479 | 0.658 | -7.351 | 0.319 |
| *OPG* | 0.403 | 7.015 | 0.618 | 0.539 | 0.729 | -7.414 | 0.269 |
| *SLAMF1* | -0.059 | 0.583 | -0.551 | 0.584 | 0.764 | -7.453 | 0.234 |
| *DNER* | -0.046 | 0.044 | -0.550 | 0.585 | 0.764 | -7.454 | 0.233 |
| *IL13* | -0.021 | 0.390 | -0.529 | 0.599 | 0.764 | -7.465 | 0.223 |
| *GDNF* | -0.041 | 1.351 | -0.520 | 0.605 | 0.764 | -7.470 | 0.218 |
| *IL4* | -0.020 | 0.232 | -0.518 | 0.607 | 0.764 | -7.471 | 0.217 |
| *CCL28* | -0.088 | 0.293 | -0.486 | 0.628 | 0.781 | -7.487 | 0.202 |
| *IL.17C* | -0.022 | 0.377 | -0.468 | 0.642 | 0.787 | -7.496 | 0.193 |
| *IL.15RA* | -0.028 | 0.027 | -0.446 | 0.657 | 0.796 | -7.506 | 0.182 |
| *IL.22.RA1* | -0.010 | 2.173 | -0.387 | 0.700 | 0.836 | -7.531 | 0.155 |
| *CST5* | -0.159 | 1.200 | -0.351 | 0.727 | 0.856 | -7.545 | 0.138 |
| *FGF.5* | -0.016 | 0.015 | -0.336 | 0.738 | 0.856 | -7.550 | 0.132 |
| *IL.2RB* | -0.013 | 1.243 | -0.325 | 0.746 | 0.856 | -7.553 | 0.127 |
| *IL.17A* | -0.025 | 0.036 | -0.296 | 0.769 | 0.856 | -7.563 | 0.114 |
| *FGF.21* | -0.129 | 0.524 | -0.292 | 0.771 | 0.856 | -7.564 | 0.113 |
| *IL10* | -0.047 | 0.308 | -0.291 | 0.772 | 0.856 | -7.564 | 0.112 |
| *TSLP* | 0.013 | 0.996 | 0.264 | 0.792 | 0.866 | -7.571 | 0.101 |
| *NT.3* | -0.008 | 0.786 | -0.255 | 0.800 | 0.866 | -7.574 | 0.097 |
| *CCL25* | -0.014 | 0.013 | -0.227 | 0.822 | 0.879 | -7.581 | 0.085 |
| *NRTN* | -0.003 | 0.003 | -0.212 | 0.833 | 0.881 | -7.584 | 0.079 |
| *CCL11* | 0.050 | 0.256 | 0.190 | 0.850 | 0.888 | -7.589 | 0.071 |
| *TGF.alpha* | -0.002 | 0.002 | -0.173 | 0.863 | 0.892 | -7.592 | 0.064 |
| *CX3CL1* | -0.002 | 0.627 | -0.146 | 0.884 | 0.904 | -7.596 | 0.053 |
| *LIF.R* | 0.000 | 0.000 | 0.000 | 1.000 | 1.000 | -7.607 | 0.000 |
| *Beta.NGF* | 0.000 | 0.278 | 0.000 | 1.000 | 1.000 | -7.607 | 0.000 |

*Please refer to Table S1 for an explanation of the abbreviations.*
